# Clinicopathologic features and integrated outcome analysis of pediatric and young adult salivary secretory carcinoma

**DOI:** 10.3389/fonc.2026.1820308

**Published:** 2026-06-15

**Authors:** Liu Yang, Dianying Liao, Wen Li

**Affiliations:** 1 Department of Otolaryngology Head and Neck Surgery, West China Hospital, Sichuan University, Chengdu, China; 2 Sichuan University West China Hospital Department of Pathology, Chengdu, China

**Keywords:** clinical outcomes, clinicopathologic analysis, pediatric salivary gland tumors, salivary secretory carcinoma, surgical management

## Abstract

**Background:**

Salivary secretory carcinoma (SSC) is a rare salivary gland malignancy characterized by recurrent ETV6-related gene fusions and generally classified as low grade. However, age-specific outcome data in pediatric and young adult patients remain limited. The present study aimed to characterize clinicopathologic features and clinical outcomes of SSC in younger patients and to contextualize these findings through an integrated analysis of published cases.

**Methods:**

We retrospectively analyzed 35 patients with SSC treated at a single institution between 2019 and 2023, including 11 individuals aged ≤29 years. Clinical, histopathologic, immunohistochemical, molecular, and outcome data were evaluated. A focused literature search identified 47 eligible publications comprising 83 reported pediatric and young adult cases, which were incorporated into a descriptive integrated analysis.

**Results:**

all younger patients presented with parotid tumors and exhibited a higher median Ki-67 labeling index compared with older patients (15.0% vs 7.5%). Among 50 evaluable published cases, recurrence occurred in 9 patients, corresponding to a pooled recurrence rate of 18.0% (95% CI 7.4–28.6%). Lymph node metastasis was documented in 10.0% of evaluable cases. The recurrence rate observed in our young subgroup (18.2%) was consistent with pooled estimates.

**Conclusions:**

SSC in pediatric and young adult patients may demonstrate a broader biological spectrum than traditionally assumed. Although generally considered low grade, a measurable subset of younger patients exhibits recurrent or metastatic behavior, underscoring the importance of systematic outcome assessment in this population.

## 1 Introduction

Salivary secretory carcinoma (SSC) is a rare malignancy originally described as mammary analogue secretory carcinoma because of its striking morphologic and molecular resemblance to secretory carcinoma of the breast. It was first proposed and defined by Skalova in 2010 (1). Later studies have established SSC as a distinct pathological entity characterized by ETV6-related gene fusions, most commonly ETV6::NTRK3 fusions. In recent years, the gene fusion deletion has also been revealed (2, 3). Because of its reproducible molecular signature and clinicopathologic features, SSC has been formally recognized as a separate tumor entity in the World Health Organization classification of head and neck tumors in 2017. The parotid gland represents the most frequent site of origin, though SSC may also arise in other major and minor salivary gland (4).

Despite its well-defined molecular background, the diagnosis of SSC remains challenging in routine clinical practice. Histologically, SSC shows a broad morphologic spectrum, including microcystic, solid, papillary, and cribriform growth patterns with intraluminal eosinophilic secretions and considerable overlapping of other salivary gland neoplasms such as acinic cell carcinoma (3, 5). Immunohistochemistry of tumor cells show strong positivity for mammaglobin, DOG1 and S-100 protein, while negativity for the myoepithelial markers such as P63, Calponin and SMA. Pan-TRK is commonly used as a screening tool for potential NTRK fusions, despite of known limitations (6). Genetic testing is usually required to establish the diagnosis. Most reports indicate that SSC presents as a well-defined entity with coexisting cystic and solid components on imaging but it is far from being a diagnostic basis.

It is reported that SSC has a relatively higher incidence than other parotid benign and malignant tumors in children and younger adults (7, 8). This may be attributed to its rarity and late released diagnostic criteria. As a result, SSC in children, adolescents, and young adults is at risk of being misclassified, delayed in diagnosis, or subjected to extended diagnostic workup. An increasing number of pediatric and young adult isolated SSC cases or small case series have been reported with younger patients often embedded within larger adult cohorts. Systematic clinicopathologic analyses focusing specifically on age-related differences, diagnostic workflows, and real-world clinical outcomes in younger patients remain scarce.

A retrospective analysis of a single-institution cohort of SSC was conducted, with particular emphasis on pediatric and young adult patients. Patients were classified into three age groups (<20 years, 20–29 years, and ≥30 years) to facilitate age-based comparison. In addition to clinicopathologic characterization, this cohort includes the pediatric case with disease recurrence and extended long-term follow-up, supported by comprehensive radiologic, pathologic, and clinical documentation. The aim is to characterize the histologic spectrum and anatomic distribution of SSC across age groups, to identify common diagnostic pitfalls encountered in younger patients, and to assess available clinical outcomes. By integrating longitudinal outcome data with practical diagnostic considerations, this study also aims to provide insights that may facilitate timely diagnosis and long-term management of SSC in pediatric and young adult.

## 2 Patients data and methods

### 2.1 Study design and clinical data collection

This retrospective, single-institution cohort study was conducted at West China Hospital, Sichuan University. Cases of SSC were identified by searching the institutional pathology archives between July 2019 and December 2023. Eligible cases included primary SSC arising in major or minor salivary glands with available histologic material for review. Cases were excluded if the clinical records are incomplete or the patients lost follow up. Patients were initially stratified into three age groups: <20 years, 20–29 years, and ≥30 years to describe the age distribution of SSC across pediatric, young adult, and older adult populations. Because truly pediatric cases were limited, comparative analyses of clinical characteristics, treatment-related variables, and follow-up outcomes were performed using a dichotomized grouping of younger patients (≤29 years) versus older patients (≥30 years). This dichotomized age grouping was intended for exploratory descriptive comparison under the constraints of a small rare-disease cohort and was not intended to define all patients aged ≤29 years as pediatric.

Demographic and clinical variables were extracted from electronic medical records, including age at diagnosis, sex, tumor duration (defined as symptom onset to diagnosis/first exposure to surgery), tumor location, fine-needle aspiration (FNA) results, preoperative imaging and treatment-related variables (extent of surgery and neck dissection). Tumor size was recorded as the greatest dimension measured on imaging and categorized as <2 cm, 2–4 cm and >4 cm groups. Pathologic lymph node stage (pN) of the patients who underwent neck dissection was assigned according to the 8^th^ AJCC/UICC TNM staging system. To facilitate clinical comparison, analyses were performed using both the three predefined age strata (**Table 1**) and a dichotomized age grouping (young ≤29 years vs older≥30 years) (**Tables 2**–**4**) that consistent with the study tables. Imaging signs of malignancy were defined as preoperative presence of at least one radiologic feature suggestive of malignancy (e.g., ill-defined/infiltrative margins, perineural spread along vertical segment of facial nerve, skin invasion or radiologically suspicious of cervical lymph node metastasis) within 1 week before surgery. Treatment information including available neck dissection protocol and adjuvant therapy was collected. The follow-up duration is counted from the initial surgery to the last follow-up or death. Recurrence was defined as local, regional, or distant metastasis after initial surgery based on clinical, radiologic, and/or pathologic confirmation.

**Table 1:** Cohort summary.

Characteristic	<20 years	20–29 years	≥30 years
N	5	6	24
Age, years (median, IQR)	14.0 (11.2–16.5)	25.0 (25.0–25.8)	48.5 (44.5–55.0)
Age range	9–18	25–28	33–79
Parotid-region	5 (100%)	6 (100%)	15 (63%)
Submandibular	0 (0%)	0 (0%)	3 (12%)
Oral/Palate/minor salivary gland	0 (0%)	0 (0%)	6(25%)

**Table 2:** Comparison of clinical and treatment-related characteristics between young and older patients with SC.

Characteristic	Young group (≤29 years)	Older group (≥30 years)	P value	Effect size
Tumor duration, years (median, IQR)	2 (2–3)	3 (1.5–4.5)	0.436	HL Δ -0.5 (-2–1) (n=11/24)
Preoperative FNA performed, n/N	5/11	12/24	1.000	0.83 (0.20–3.49)
Male sex, n/N	8/11	15/24	0.709	1.60 (0.34–7.64)
Imaging signs of malignancy, n/N	3/11	6/24	1.000	1.12 (0.22–5.67)
Tumor size group, n/N			0.783	Cramer’s V = 0.15
— <2 cm	1/11	5/24		
— 2–4 cm	7/11	13/24		
— >4 cm	3/11	6/24		
Neck dissection performed, n/N	5/11	11/24	1.000	0.98 (0.23–4.13)
Lymph Node stage (n/N)			0.471	Cramer’s V = 0.38
pN0	3/5	8/11		
pN1	1/5	3/11		
pN2	1/5	0/11		

**Table 3:** Key IHC and molecular findings.

Marker	Young tested (n/N)	Young positive (n/tested)	Overall tested (n/N)	Overall positive (n/tested)
CK7	11/11	11/11	25/35	25/25
S-100	11/11	10/11	32/35	31/32
SOX10	3/11	3/3	7/35	7/7
GATA3	0/11	—	6/35	3/6
pan-TRK	10/11	10/10	19/35	18/19
p63	10/11	0/10	29/35	3/29
p40	2/11	0/2	4/35	0/4
DOG1	3/11	0/3	13/35	0/13
ETV6	10/11	4/10	29/35	16/29
Ki-67 (%)	10/11	median 15.0 (5.0–20.0)	21/35	median 7.5 (5.0–18.8)

**Table 4:** Follow-up and outcomes by age group in SC.

Characteristic	Young group (≤29 years)	Older group (≥30 years)	P value	Effect size
Permanent Postoperative Facial Paralysis^*^	1/11	3/24	1.000	0.70 (0.06–7.60)
Follow-up, months (median, IQR)	36 (30–42)	24 (12–54)	0.456	HL Δ 0 (-12–24) (n=11/24)
Recurrence n/N	2/11	2/24	0.575	2.44 (0.30–20.12)
Outcome, live	10/11	22/24	1.000	0.91 (0.07–11.23)

* Permanent facial paralysis was defined as the facial paralysis symptoms did not recover more than 2 years after surgery or did not recover until the last follow-up or death.

### 2.2 Treatment strategy (institutional surgical principles)

For the tumors of parotid gland, the surgical approach was determined according to the suspected malignant potential. When malignancy was suspected based on preoperative fine-needle aspiration cytology, imaging, and/or intraoperative frozen-section assessment, total parotidectomy (or extended parotidectomy) with neck dissection of levels II–III was performed, with extension to levels IV–V when clinically warranted. In the absence of suspected malignancy, superficial, partial or deep parotidectomy was applied, which depends on the tumor’s location. For tumors arising outside the parotid gland, complete local excision of the lesion with negative frozen margins was applied, additional procedures including neck dissection were performed when clinically indicated.

### 2.3 Pathologic review and diagnostic criteria

**Figure 1:**
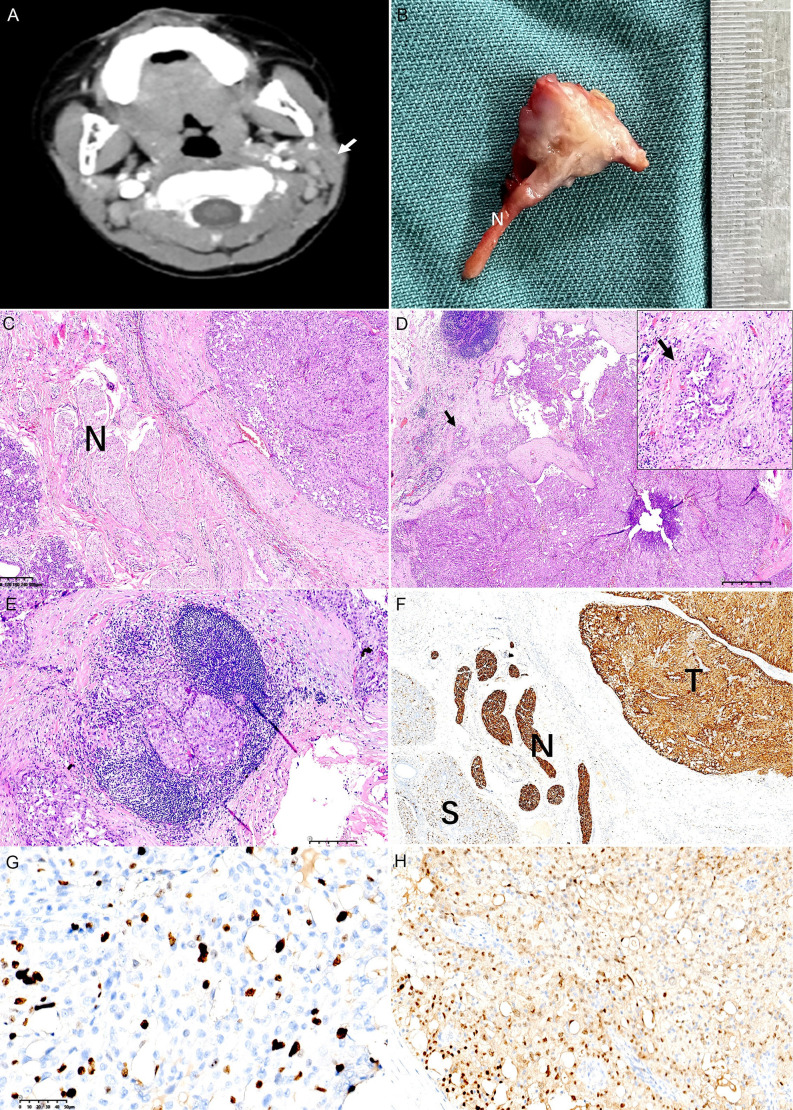
Typical case of SSC with 1 year history and prominent capsular invasion in the parotid gland of a 9-year-old child. **(A)** A small ill-defined tumor 1.2cm×1.0cm×0.8cm in dimension located in the caudal part of the parotid gland where the main stem of great auricular nerve penetrated (white arrow); **(B)** The cross section of the tumor demonstrated the penetration of the great auricular nerve(N) which resulted in the sacrifice of the nerve; **(C)** The interface of the tumor, nerve(N) and parotid gland demonstrated close adhesion between the nerve and the tumor, the dense capsule and indistinct capsular invasion(HE staining, original magnification ×60); **(D)** Obvious capsular invasion and scattered cancer cell nest (black arrow) in the capsule and its zoom-in image(inset) (HE staining, original magnification ×30, inset: ×100); **(E)** Cancer nest in the hyperplastic lymph node of the capsule (HE staining, original magnification ◊100); **(F)** The tumor(T), nerve(N)and salivary gland cells(S) demonstrated moderate to strong positivity of S100 (immunohistochemistry, IHC, ×40); **(G)** The tumor cells revealed about 20% Ki67 labelling index (immunohistochemistry, IHC, ◊400); **(H)** The tumor cells revealed strong positivity for TRK (pan) which supported the diagnosis of SSC and indicated for a targeted therapy in those recurrent or late- stage patient.

All available pathological slides were re-reviewed by two head and neck pathologists to confirm the diagnosis and evaluate histologic patterns. The diagnosis of SSC was rendered based on characteristic morphology and immunophenotype, with molecular confirmation when available. The Immunohistochemistry (IHC) panel including CK7, S-100, SOX10, GATA3, pan-TRK, p63, p40, and DOG1 was selected to establish the diagnosis and to exclude a few key mimics (e.g., acinic cell carcinoma and myoepithelial neoplasms). The Ki-67 index was evaluated in hotspots by counting at least 500–1000 tumor cells and reported as a percentage. Molecular testing for ETV6-related rearrangement was performed in a subset of cases. A typical case of SSC with prominent feature of capsular invasion in the parotid gland of a 9-year-old child was affiliated (**Figure 1**).

### 2.4 Integrated literature analysis

To contextualize the findings of our institutional cohort, a focused literature review was conducted to identify published cases of salivary secretory carcinoma (SSC) in pediatric and young adult patients (≤29 years). PubMed was searched for articles published between January 2010 and March 2025 using the terms “secretory carcinoma,” “mammary analogue secretory carcinoma,” “salivary,” “pediatric,” “child,” “adolescent,” and “young adult”.

Studies were eligible if they reported individual or aggregated data on pediatric or young adult SSC cases with available clinical outcome information. Review articles were screened to avoid duplicate case inclusion. When multiple reports described overlapping cohorts, only the most complete dataset was included.

Data extracted from published reports included age, tumor site, lymph node status, recurrence, follow-up duration, immunohistochemical findings, molecular testing results, and Ki-67 index when available. Because reporting of immunohistochemical panels, molecular assays, Ki-67 index, and follow-up endpoints was heterogeneous and incomplete across the published literature, these variables were summarized descriptively and were not subjected to formal pooled statistical analysis. Because of heterogeneity in reporting and incomplete availability of individual-level data, a formal meta-analysis was not performed. Instead, a descriptive integrated analysis was conducted by combining the published cases with the present cohort.

Pooled proportions were calculated using available-case analysis, with denominators reflecting only cases in which the specific variable was reported.

### 2.5 Statistical analysis

Continuous variables are presented as median (IQR) and compared using the Mann–Whitney U test (two groups) or Kruskal–Wallis test (three groups), as appropriate. Categorical variables are presented as n/N and compared using Fisher’s exact test; for R×C tables, the Fisher–Freeman–Halton exact test was applied when appropriate. Effect sizes are reported as odds ratios (ORs) with 95% confidence intervals (CIs) for binary variables, Hodges–Lehmann (HL) location shift estimates with 95% CIs for continuous variables, and Cramer’s V for multi-category variables. Two-sided P values <0.05 were considered statistically significant; given the limited sample size, P values were interpreted as exploratory. Analyses were performed using SPSS (version 29.0; IBM Corp.).

### 2.6 Ethics statement

This study was approved by the Medical Ethics Committee of West China Hospital, Sichuan University (approval No. 20221106). Given the retrospective study and use of de-identified data, the requirement for informed consent was exempted. The study was conducted in accordance with the Declaration of Helsinki and its later amendments.

## 3 Results

### 3.1 Cohort characteristics and anatomic distribution

A total of 35 salivary gland–region secretory carcinomas (SC) were included. By age strata, there were 5 patients <20 years, 6 patients aged 20–29 years, and 24 patients ≥30 years (**Table 1**). Median ages were 14.0 (IQR 11.2–16.5), 25.0 (25.0–25.8), and 48.5 (44.5–55.0) years, respectively. SSCs most commonly located in the parotid gland: all cases in the <20 and 20–29 groups were parotid tumors (100% each), whereas in the ≥30 group, 63% were parotid tumors, with the remainder in the submandibular gland (12%) and oral/palate/minor salivary glands (25%).

### 3.2 Clinical presentation and preoperative evaluation

Most patients presented with a slowly growing, painless mass in the parotid region on admission. One patient (in young group) with recurrent disease was an exception, presenting with peripheral facial palsy and a rapidly enlarging facial mass accompanied by numbness of the overlying skin in the affected area. Given the limited number of truly pediatric patients, subsequent comparisons of clinical features, treatment variables, and outcomes were performed between younger patients aged ≤29 years and older patients aged ≥30 years. In the dichotomous age comparison, the median cancer duration was 2 years (IQR 2 to 3) in the younger group and 3 years (IQR 1.5 to 4.5) in the older group (P = 0.436; HLΔ =-0.5 years, 95%CI -2 to 1; **Table 2**). The proportion of FNA performed before operation was similar between the two groups (5/11 vs 12/24), and the proportion of males was also similar (8/11 vs 15/24). There was also no significant difference between the two groups in the rate of radiographic malignancy (3/11 vs 6/24; OR 1.12, 95%CI 0.22-5.67; **Table 2**). The most common tumor size was 2–4 cm (7/11 in the young group; 13/24 in the elderly group), and there was no significant difference in the overall distribution (P = 0.783; Cramer’s V = 0.15).

### 3.3 Neck dissection and nodal status

Neck dissection was performed at similar frequencies (5/11 in the young group and 11/24 in the older group; OR 0.98, 95%CI 0.23–4.13; **Table 2**). Among patients who underwent neck dissection, there was no statistically significant difference in nodal stage distribution (P = 0.471; Cramer’s V = 0.38): pN0/pN1/pN2 were 3/5, 1/5, and 1/5 in the young group versus 8/11, 3/11, and 0/11 in the older group (**Table 2**).

### 3.4 Immunohistochemistry and molecular findings

Immunohistochemistry demonstrated a characteristic SC profile: CK7 was positive in all tested cases (11/11 in the young group; 25/25 overall), S-100 was frequently positive (10/11 in the young group; 31/32 overall), and pan-TRK showed a high positivity rate (10/10 in the young group; 18/19 overall). In contrast, p63/p40 and DOG1 were largely negative (young: p63 0/10, p40 0/2, DOG1 0/3; overall DOG1 0/13). ETV6-related testing was performed in a subset, with positivity in 4/10 tested young cases and 16/29 tested cases overall. The median Ki-67 index was 15.0% (5.0–20.0) in the young group and 7.5% (5.0–18.8) overall (**Table 3**).

### 3.5 Follow-up and outcomes

The median follow-up was 36 months (IQR 30–42) in the young group and 24 months (IQR 12–54) in the older group (P = 0.456; HL Δ=0 months, 95%CI -12 to 24; **Table 4**). Permanent postoperative facial paralysis occurred in 1/11 young patients and 3/24 older patients (OR 0.70, 95%CI 0.06–7.60). One pediatric patient and one elderly patient with lymph node metastasis received radiotherapy. Recurrence was observed in 2/11 young patients and 2/24 older patients (OR 2.44, 95%CI 0.30–20.12). At last follow-up, most patients were alive (10/11 in the young group and 22/24 in the older group; OR 0.91, 95%CI 0.07–11.23).

Given the rarity of SSC and limited sample size, age-stratified comparisons were exploratory; effect estimates with 95% confidence intervals are provided to reflect uncertainty. Cases with shorter follow-up were retained to avoid additional selection bias in this rare disease cohort.

### 3.6 Integrated analysis of published pediatric and young adult SSC

**Figure 2:**
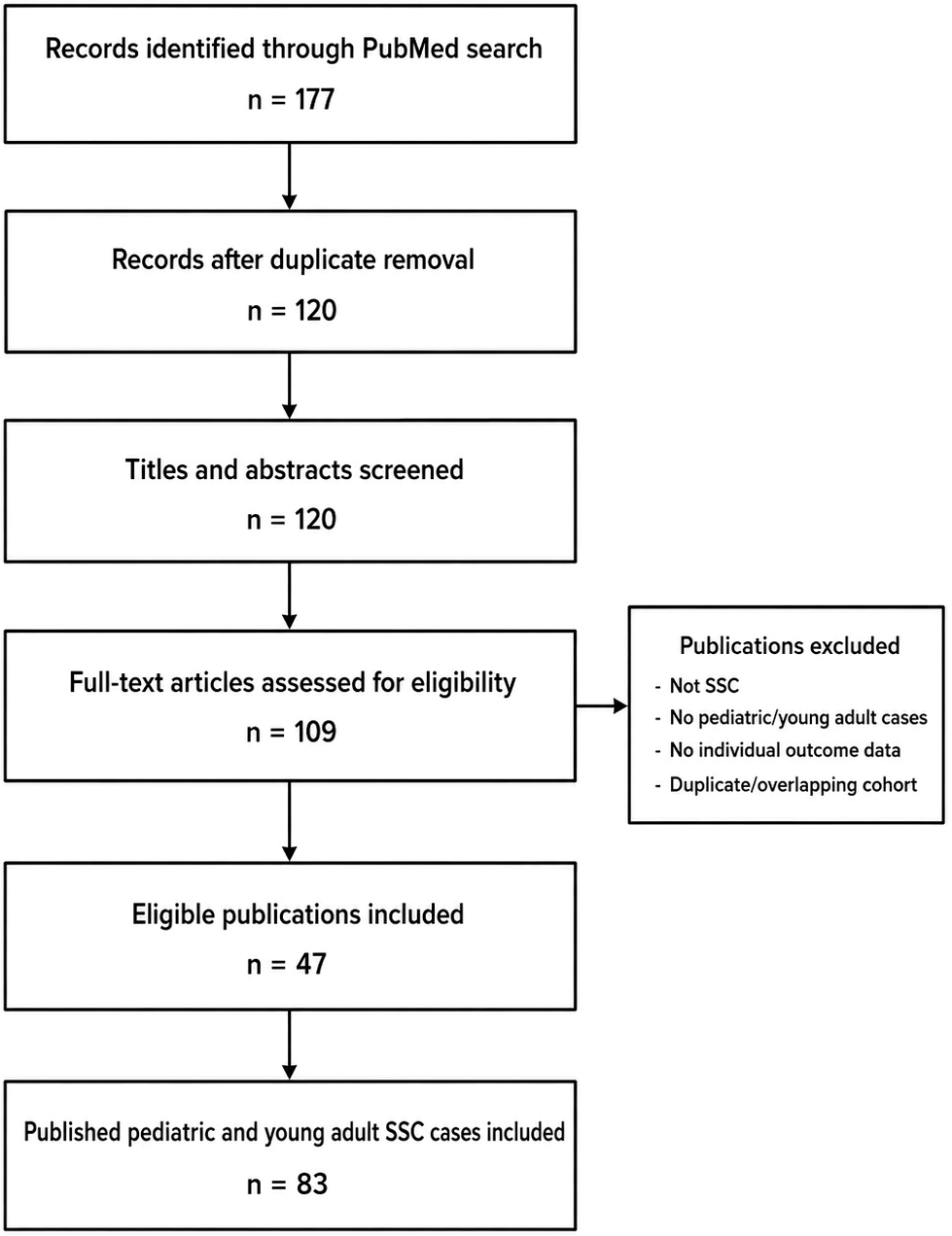
Flow diagram of the literature search and case selection process for the integrated analysis of published pediatric and young adult salivary secretory carcinoma cases.

The literature search identified 177 records through database searching. After removal of duplicates, 120 titles and abstracts were screened. Following full-text review, 47 publications met the eligibility criteria and were included in the integrated analysis, yielding a total of 83 pooled pediatric and young adult cases (**Supplementary Table 1**) (7, 9–52). The literature review yielded 83 published pediatric and young adult cases (**Figure 2**). These were analyzed separately and compared with the 11 young patients from our institutional cohort. Tumor site was reported in all cases, with the parotid gland accounting for 64 of 83 tumors (77.1%), confirming its predominance in pediatric and young populations. Recurrence status was available in 50 cases. Recurrence was documented in 9 patients, yielding a pooled recurrence rate of 18.0% (95% CI 7.4–28.6%). Lymph node status was reported in 30 cases, with metastasis observed in 3 patients (10.0%). Notably, recurrence occurred in 2 of 11 young patients (18.2%) in our cohort, closely aligning with the pooled estimate from the literature. Due to heterogeneous reporting, pooled proportions were calculated using available-case analysis (**Table 5**). Reporting of diagnostic ancillary studies among the published cases was heterogeneous. Most reports described at least partial immunohistochemical evaluation, commonly including S-100, mammaglobin, SOX10, DOG1, and/or pan-TRK, but the exact antibody panels varied substantially across studies. Molecular confirmation was also inconsistently reported, particularly in earlier publications in which SSC was initially described as mammary analogue secretory carcinoma. Ki-67 index was available in only a minority of published cases and was therefore not suitable for pooled comparison. Accordingly, immunohistochemical, molecular, and proliferative data from the literature were interpreted descriptively rather than quantitatively.

**Table 5:** Summary of published pediatric and young adult SSC cases.

Variable	Available cases	Events	Rate (%)
Parotid	83	64	77.1
LN metastasis	30	3	10.0
Recurrence	50	9	18.0

## 4 Discussion

Pediatric salivary malignancy is uncommon, accounting for only a small fraction of childhood tumors, and salivary gland tumors overall are infrequent in patients younger than 18 years (53). Salivary Secretory carcinoma (SSC) is now well established as a distinct entity in the current WHO classification and is characterized by recurrent, tumor-defining gene fusion most commonly involving ETV6 and NTRK3 supporting the central role of molecular diagnostics in classification and clinical decision-making (54). Notably, although SSC is often considered a low-grade malignancy, published series and systematic reviews indicate that SSC spans a wide age range that includes children and adolescents, highlighting that SSC should be taken into account in the differential diagnosis for salivary gland masses in young patients (55). In this single-center cohort of 35 patients, nearly one third were younger than 29 years old. Stratified comparisons were employed to answer an important clinical question: whether or not SSC is necessarily indolent in young patients and routine preoperative evaluation can reliably guide the surgical decisions. Because SSC is rare and the limited sample size, it is not surprising that most comparisons did not reach statistical significance, so we put more emphasis on effect sizes and confidence intervals to present uncertainty and identify clinical signals worthy of further validation.

SSC is generally regarded as a low-grade malignancy; however, recurrence and nodal metastasis have been documented (56–58). In our cohort, recurrence occurred in 2 of 11 younger patients and 2 of 24 older patients, and although the confidence interval was wide, these findings indicate that young age does not ensure indolent behavior. Notably, one young patient presented with rapid progression and facial nerve involvement, underscoring the potential for aggressive disease. Our integrated analysis further demonstrated a pooled recurrence rate of approximately 18% among pediatric and young adult patients, consistent with the recurrence rate observed in our cohort. Given that the published literature largely consists of isolated case reports and small retrospective series, publication bias toward clinically unusual or aggressive pediatric cases is likely. Therefore, pooled recurrence estimates should be interpreted cautiously and viewed as hypothesis-generating rather than population-level incidence estimates. Although lymph node metastasis was less frequent (approximately 10% of evaluable cases), these findings suggest that SSC in younger patients cannot be considered uniformly benign progress. Comprehensive diagnostic evaluation, including immunohistochemical and molecular testing, along with appropriate surgical management and long-term surveillance, should therefore be recommended irrespective of age. Recurrence was observed in a subset of reported pediatric and young adult cases, indicating that age alone should not guide treatment de-escalation.

A challenge in SSC management is that preoperative assessment may underestimate malignant potential. The imaging features of secretory carcinoma often lack specificity, and it usually shows a well-defined lesion with mixed solid and cystic components on CT/MRI. It should be aware this feature is not present in most pleomorphic adenoma. MR Studies have reported that SC (formerly known as MASC) has predominantly cystic areas accompanied by solid/papillary projections. The so-called “imaging signs of malignancy” were present in 3/11 younger patients and 6/24 older patients (OR 1.12, 95% CI 0.22–5.67), suggesting that imaging-based suspicion alone did not meaningfully distinguish risk between age groups in this cohort (20, 59). FNA also has pitfalls in diagnosing SSC because its cytomorphology overlapping with benign and low-grade malignant salivary neoplasms. Molecular confirmation of ETV6::NTRK3 is often considered the diagnostic gold standard when morphology and immunophenotype are not definitive (60). Pan-TRK immunohistochemistry may serve as a useful screening tool, but published evidence indicates that sensitivity can be reduced for some NTRK3-fusion tumors, supporting confirmatory testing by FISH/RT-PCR/NGS when clinical suspicion persists despite negative screening. Besides, ETV6-negative cases have been well documented (2, 61).

Given these diagnostic limitations, surgical management should be risk-adapted according to the suspected malignant potential. This pragmatic approach reflects real-world decision-making but introduces confounding by indication when comparing treatment patterns across age groups. This risk-adapted approach is clinically pragmatic but introduces confounding by indication in retrospective analyses: surgical intensity is partly a consequence of preoperative suspicion, which itself is imperfect. Therefore, observed similarities (or differences) in procedures between age strata should be interpreted cautiously, and future multicenter studies may benefit from standardized decision algorithms and prospective documentation of the rationale for surgical extent.

Higher median Ki-67 labeling index in the younger group (15.0%, IQR 5.0–20.0) was observed in comparison with the overall cohort (7.5%, IQR 5.0–18.8). This finding should be interpreted as hypothesis-generating rather than definitive evidence of age-related biological aggressiveness. Ki-67 in salivary gland tumors is sensitive to sampling and hotspot selection, and its prognostic significance in SSC specifically remains incompletely defined. Nonetheless, in the context of a young patient with atypical clinical features (rapid growth, pain, facial nerve symptoms) or adverse histology, a higher proliferative index may support closer surveillance and multidisciplinary discussion, rather than de-escalation solely on the basis of young age.

The role of adjuvant therapy in SSC remains uncertain because available evidence largely derives from small retrospective cohorts and case-based experiences. Pediatric guidance in salivary gland tumors generally reserves radiotherapy for high-risk features such as nodal disease, positive margins, extracapsular extension, or perineural invasion, and highlights the importance of balancing disease control with long-term toxicity (62). SSC is enriched for actionable NTRK fusions, and consensus guidance recognizes NTRK inhibitors as a valuable option for disseminated, unresectable, recurrent, or metastatic SSC with ETV6–NTRK3 fusion. Clinical reports further document rapid and durable responses to first-generation TRK inhibitors such as entrectinib in recurrent/metastatic NTRK fusion–positive SSC, supporting molecular testing not only for diagnosis but also for therapeutic planning in advanced stage (63, 64). In this cohort, age-stratified comparisons did not demonstrate statistically significant differences in outcomes, and the confidence intervals around effect estimates were wide. This “negative” statistical landscape should not be interpreted as evidence of equivalence; rather, it reflects limited power and substantial uncertainty. Clinically, the recurrence observed in a pediatric patient who also received postoperative radiotherapy in the setting of nodal metastasis underscores that adjuvant treatment may not fully overcome high-risk tumor biology in selected cases, and that recurrence risk remains non-zero even with intensified therapy.

The published pediatric and young adult SSC literature also highlights important diagnostic heterogeneity. Earlier reports frequently used the terminology “mammary analogue secretory carcinoma” and often relied on morphology and limited immunohistochemical panels, whereas more recent reports increasingly incorporated pan-TRK immunohistochemistry and molecular confirmation of ETV6-related fusions. This temporal evolution in diagnostic practice limits direct comparison across published cases. In particular, incomplete molecular testing and inconsistent reporting of Ki-67 or immunohistochemical panels may have affected case ascertainment and outcome interpretation in the integrated analysis. Therefore, the literature-derived data should be regarded as contextual and hypothesis-generating rather than equivalent to a uniformly diagnosed multicenter cohort.

The integrated analysis of published pediatric and young adult SSC cases provides useful context for the present institutional cohort. Among the 83 published cases included in this review, the parotid gland was the predominant site, accounting for 77.1% of reported tumors. This finding is consistent with our institutional younger subgroup, in which all patients aged ≤29 years presented with parotid-region tumors. Although SSC is generally regarded as a low-grade malignancy, the literature-derived data also demonstrate that recurrence and nodal metastasis are not negligible among reported pediatric and young adult cases. Recurrence was documented in 9 of 50 evaluable published cases, and lymph node metastasis was reported in 3 of 30 evaluable cases. These observations are broadly consistent with our cohort, in which recurrence occurred in 2 of 11 younger patients. Nevertheless, these literature-derived estimates should be interpreted cautiously because the available cases were mainly derived from case reports and small series, with heterogeneous diagnostic confirmation, incomplete molecular testing, variable follow-up duration, and likely publication bias toward unusual or clinically aggressive cases.

This study is limited by the retrospective single-center design: the small sample size leads to insufficient power. Treatment was not randomized and influenced by the degree of preoperative suspicion (mixed indications). Molecular confirmation was not available for all cases, which represents an important limitation because ETV6-related fusion testing is central to the contemporary diagnosis of SSC. Some earlier cases were diagnosed based on characteristic morphology and immunophenotype in the context of real-world clinical practice. The ≤29-year cutoff used in **Tables 2**-**4** should be interpreted as an exploratory younger-patient grouping rather than a standard pediatric definition. This approach was adopted because the number of truly pediatric patients was too small to support meaningful separate comparative analysis. Nevertheless, this cohort still presents a more realistic clinical management situation of SSC in different age groups, highlights the decision nodes caused by imaging/FNAC uncertainty, and records the fact that “young age does not exclude recurrence”. Future studies should improve the precision of effect size estimation through multicenter pooling, standardize the definition of “high-risk SSC” (including proliferative activity and adverse pathological features), and further clarify the indications for neck dissection and adjuvant therapy in pediatric/young adult patients. Therefore, the present study should primarily be interpreted as a descriptive exploratory case series with integrated literature contextualization rather than a definitive outcome study.

## 5 Conclusions

In summary, SSC should be considered in the differential diagnosis of pediatric and young adult salivary gland tumors. Diagnostic limitations of imaging and FNA cytology can complicate risk assessment and surgical planning. Age-stratified comparisons were exploratory, the observed recurrence signs and higher Ki-67 in younger patients suggest that careful long-term follow-up is warranted and that young age alone should not drive de-escalation.

## Data Availability

The original contributions presented in the study are included in the article/**Supplementary Material**. Further inquiries can be directed to the corresponding author.
